# Optimised phycoerythrin extraction method from *Porphyridium* sp. combining imidazolium-based ionic liquids

**DOI:** 10.1016/j.heliyon.2024.e34957

**Published:** 2024-07-20

**Authors:** Alejandro Piera, Juan J. Espada, Victoria Morales, Rosalía Rodríguez, Gemma Vicente, Luis Fernando Bautista

**Affiliations:** a Department of Chemical and Environmental Technology. ESCET, Universidad Rey Juan Carlos. Tulipán S/n, 28933, Móstoles, Madrid, Spain; b Department of Chemical, Energy and Mechanical Technology. ESCET, Universidad Rey Juan Carlos. Tulipán S/n, 28933, Móstoles, Madrid, Spain; c Instituto de Tecnologías para la Sostenibilidad, Universidad Rey Juan Carlos. Tulipán S/n, 28933, Móstoles, Madrid, Spain

**Keywords:** Phycoerythrin, Ionic liquids, *Porphyridium* sp., Ultrasound-assisted extraction, Response surface methodology, Microalgae

## Abstract

Phycoerythrin (PE) extraction from *Porphyridium* sp. was studied employing ultrasound-assisted extraction combined with aqueous mixtures of two imidazolium-based ionic liquids (ILs) simultaneously, marking a significant novelty. A face-centred central composite design and response surface optimised PE yield (E_PE_), considering the effects of ionic liquid concentration (IL), [Emim][EtSO_4_]/[Bmim][EtSO_4_] mass ratio (E/B), biomass concentration (BM), and time (t). Improvements in E_PE_ by 300 % and 115 % were achieved compared to a phosphate buffer solution and the freeze-thaw method, respectively. Temperature and pH effects were examined independently, leading to the determination of optimal operating conditions: BM = 10 mg mL^−1^, IL = 18.6 wt%, E/B = 0.78/0.22, t = 10 min, T = 35 °C, and pH = 7.5. Results indicated the potential for reusing the ILs for at least five consecutive extraction cycles, maintaining an E_PE_ of 94.2 % compared to fresh ones. This underscores the success and innovation of the developed technology in enhancing PE extraction from *Porphyridium* sp.

## Highlights

• Combine UAE + ILs aqueous solutions is a feasible method to extract phycoerythrin.
• 2^4^ Full factorial experimental design is proposed for phycoerythrin extraction.
• The most significant factors are concentration of ILs and biomass, following by ILs mixture.
• [EMIM][EtSO_4_]/[BMIM][EtSO_4_] mixture improve the extraction of phycoerythrin.
• Extracted amount of phycoerythrin was constant by reusing the ILs for four cycles.

## Graphical abstract

**Figure:**
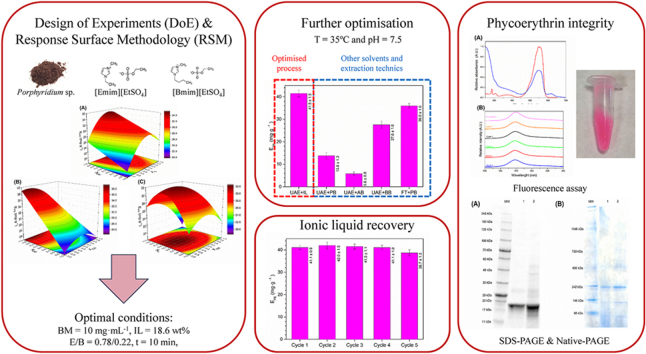

## 1 Introduction

Microalgae can accumulate a wide variety and remarkable amounts of bioproducts to be used in applications such as human health, pharmaceutical, and nutraceutical, as well as to produce biofuels [[1], [2], [3]]. *Porphyridium* is a genus of unicellular red algae belonging to the phylum Rhodophyta, the class Porphyridiophyceae, the order Porphyridiales and the family Porphyridiaceae (retrieved from the Integrated Taxonomic Information System, http://www.itis.gov) that can accumulate significant quantities of high-value bioactive products as extracellular polysaccharides (EPS), polyunsaturated fatty acids (PUFAs), sterols, vitamins, and specially phycoerythrin (PE), a kind of phycobiliprotein (PBP) [4].

PBPs are light-harvesting protein pigments present in the thylakoid membrane of chloroplasts [58]. They can be classified based on their light absorption properties: phycoerythrins (PE, λ_max_ 540–570 nm), phycocyanins (PC, λ_max_ 610–620 nm), and allophycocyanins (APC, λ_max_ 650–655 nm). Some cyanobacteria have a fourth type of biliprotein instead of PE, i.e., phycoerythrocyanin (PEC λ_max_ 570–595 nm) [5]. In turn, PE can be divided into three main classes: B-phycoerythrin (B-PE: peaks at 545, 565 nm with a shoulder at 499 nm), R-phycoerythrin (R-PE: peaks at 499, 565 nm and a shoulder at 545 nm) and C-phycoerythrin (C-PE: peak at 565 nm) [[6], [7], [8]]. PE has beneficial properties for human health based on its antioxidant and hepatoprotective activity, allowing the prevention of diseases such as cancer, diabetes, inflammation, and neurodegenerative disorders [[9], [10], [11], [12], [13], [59]]. Moreover, PE is a valuable candidate for designing and characterising light-sensing elements in biosensors and as tags of cells and macromolecules [14,15].

The recovery of PBP (and hence PE) stored in the phycobilisome requires cell wall disruption and an extraction process using solvents. These stages are commonly performed by a physical pretreatment, such as maceration or freeze/thaw to break the cell wall and the use of distilled water, salts, or buffer solutions as a solvent [6,11,[16], [17], [18], [19]]. However, these methods present some drawbacks regarding reduced extraction yields and solvent reusability.

Recently, innovative extraction techniques, called green technologies, have emerged to replace conventional extraction processes. They focus on reducing the energy consumption of the process, using green solvents, obtaining bioactive compounds, and improving extraction yield, quality, and purity of the molecules safely recovered [[20], [21], [22], [23]]. The most employed technologies to recover PE are microwave extraction (MW), ultrasound-assisted extraction (UAE), high-pressure homogenisation (HPH), supercritical-fluid extraction (SFE), and accelerated solvent extraction (ASE) (also called pressurised liquid/solvent extraction (PLE/PSE) or subcritical solvent extraction (SSE)) [[23], [24], [25]].

On the other hand, green solvents are environmentally friendly solvents developed as an alternative to petrochemical solvents [26]. The structure and chemical composition of ionic liquids are diverse, and their physicochemical and toxicological properties can vary significantly. However, many ionic liquids (ILs) are considered clean and safe solvents [27] and a promising alternative to traditional solvents for PBP extraction due to their versatility and reusability [28,29], their capabilities to disrupt the microalgae [30], and their low volatility, low toxicity, and non-flammability [31,32]. Also, they present high solvation ability, thermal and chemical stability, and the possibility of combining their ions to adjust their properties and can be regenerated [28,33]. Nevertheless, ILs may be expensive and present low biodegradability; therefore, it is essential to choose a suitable one [34]. In this regard, reported research showed that imidazolium-based ionic liquids are less toxic than other commonly containing choline, tetrabutylammonium, or pyridinium-based as cations [35,36]. In addition, among imidazolium-based ionic liquids, the toxicity increases with the length of the alkyl chain due to an increase in the lipophilicity of the cation, which, in turn, increases the interaction with cell membranes [37]. Therefore, the two cations used in our work (1-ethyl-3-methylimidazolium and 1-butyl-3-methylimidazolium) correspond to the short-chain imidazolium-based group showing a low level of toxicity among common ionic liquids.

Protein and PBP (including PE) extraction from microalgae by using aqueous solutions of some ILs have also been reported [29,[38], [39], [40], [41]]. In this sense, a wide variety of ILs are available, depending on the type of cation, anion, or length of the alkyl chain in imidazolium-based ILs, among other factors. In the PBP extraction process, the effect of diverse anions (chloride, dicyanamide, dimethylphosphate, thiocyanate, trifluoromethanesulfonate, methanosulfonate, trifluoroacetate, or acetate) has been studied [38,40]. The basicity of the anion and the ability to form hydrogen bonds is correlated. The higher the anion chain, the lower its ability to form hydrogen bonds and the lower the solubility of the target molecule in IL. Thus, anions like Cl^−^, [CH_3_CO_2_]^−^, [CH_3_SO_3_]^−^ or [EtSO_4_] − have a higher capacity to extract PBP [40,42]. Concerning IL cations, their structure and size determine the melting point and the polarizability of the ILs, directly influencing the solubilisation of the proteins [40]. Hydrophobic cations (phosphonium and ammonium-based ILs) are less effective in extracting PBP than choline and imidazolium-based ILs [40]. In the case of imidazolium-based ILs, literature shows that short chains achieve better extraction yield because as the chain length increases, the IL becomes more hydrophilic [40]. In agreement, ILs are highly recommended solvents for proteins. Although it is possible to dissolve proteins in pure ILs, their aqueous solutions are preferred because of higher solubility, increased stability (operational and thermal), and reduced costs [43]. Due to the amphiphilic character of proteins, water and ions from ionic liquids interact with areas of the protein surface, which may be polar or non-polar [44]. The present study is focused on the UAE method combined with green solvents. On the one hand, UAE is a technique based on the cavitation phenomenon, which allows an adequate mixing and high mass transfer attributed to the significant penetration of the solvent into the sample matrix and the greater surface contact area between the solid-liquid phases [45]. In this scenario, the present work aims to optimise PE extraction conditions from *Porphyridium* sp. using 1-ethyl-3-methylimidazolium ethyl sulfate ([Emim][EtSO_4_]), 1-butyl-3-methylimidazolium ethyl sulfate ([Bmim][EtSO_4_]), and their mixture combined with ultrasonication. These ILs were chosen based on several advantages, such as a simple synthesis process, low cost, and good electrochemical properties [46]. Their short alkyl-chain length and the organic nature of the anion [EtSO_4_]^-^ make both ILs an eco-friendlier alternative than other ILs [34]. In addition, high yield extraction of PBP by [Emim][EtSO_4_] and good reusability were corroborated [42]. For this purpose, a 2^4^ full factorial design was carried out to maximise the PE extraction yield (E_PE_). Four factors were studied, namely biomass concentration, total ionic liquid concentration, [Emim][EtSO_4_]/[Bmim][EtSO_4_] mass ratio and extraction time. Besides, the effect of temperature and pH were also studied to optimise the process further. All experiments employed a constant sonication frequency of 37 kHz at 80 % amplitude. Finally, the recovery of ILs at the optimal extraction conditions was carried out using dialysis. The present study is focused on the UAE method combined with green solvents. On the one hand, UAE is a technique based on the cavitation phenomenon, which allows an adequate mixing and high mass transfer attributed to the significant penetration of the solvent into the sample matrix and the greater surface contact area between the solid-liquid phases [45]. In this scenario, the present work aims to optimise PE extraction conditions from *Porphyridium* sp. using 1-ethyl-3-methylimidazolium ethyl sulfate ([Emim][EtSO_4_]), 1-butyl-3-methylimidazolium ethyl sulfate ([Bmim][EtSO_4_]), and their mixture combined with ultrasonication. The utilisation of mixtures of ILs for the particular extraction of PE from this microalga has not been previously investigated in the literature. These ILs were chosen based on several advantages, such as a simple synthesis process, low cost, and good electrochemical properties [46]. Their short alkyl-chain length and the organic nature of the anion [EtSO_4_]^-^ make both ILs an eco-friendlier alternative than other ILs [34]. In addition, high yield extraction of PBP by [Emim][EtSO_4_] and good reusability were corroborated [42]. For this purpose, a 2^4^ full factorial design was carried out to maximise the PE extraction yield (E_PE_). This approach offers several advantages, including efficiently exploring the design space, identifying nonlinear responses, evaluating the effects of factors at different levels, and facilitating process optimisation with a minimal number of experiments. Four factors were studied, namely biomass concentration, total ionic liquid concentration, [Emim][EtSO_4_]/[Bmim][EtSO_4_] mass ratio and extraction time. Besides, the effect of temperature and pH were also studied to optimise the process further. All experiments employed a constant sonication frequency of 37 kHz at 80 % amplitude. Finally, the recovery of ILs at the optimal extraction conditions was carried out using dialysis. No work in the literature has specifically targeted the use of ionic liquids for PE extraction from *Porphyridium* sp. Furthermore, none have addressed the study of the conditions and interactions relevant to this specific extraction process through an experimental design methodology, including the specific investigation of temperature and pH.

## 2 Materials and methods

### 2.1 Materials

Cianoalgae S.L. (Madrid, Spain) provided *Porphyridium* sp. as a dried powder. [Bmim][EtSO_4_] (98 %) was purchased from IoLiTec-Ionic Liquids Technologies GmbH (Heilbronn, Germany). [Emim][EtSO_4_] (95 %), [Hmim][EtSO_4_], [Omim][EtSO_4_] (95 %), ethanol, and the dialysis membrane, with a molecular weight cut-off of 14 kDa, were purchased from Sigma-Aldrich (ST Louis, MO, USA).

### 2.2 Phycoerythrin extraction

Extraction experiments were carried out by combining UAE and ILs. For this purpose, biomass (0.1, 0.3 or 0.6 g) was mixed with 10 mL of solvent, which consists of aqueous IL (or ILs mixture) solutions at different concentrations (0, 15 or 30 wt%). The mixture biomass-solvent was stirred for 30 s in a Vortex mixer and then sonicated for various periods (10, 20, or 30 min) at 25 °C by using Elmasonic P ultrasound equipment (Elma Schmidbauer GmbH, Singen, Germany) providing a power of 656 W (80 % amplitude) at a constant frequency of 37 kHz [42], within the effective frequency of ultrasound ranges for bioactive compound extraction (20–50 kHz), as reported in the [47]. The mixture was centrifuged at 5000 rpm for 10 min in an Eppendorf centrifuge 5910 (Hamburg, Germany) to separate the supernatant (rich in PE) from the waste biomass. The extraction yield (E_PE_) of PE was measured by spectrophotometric absorption (UV–visible NanoDrop™ 2000/2000c) using adapted equations (1), (2), (3)) from previous studies [16,48], as follows:

(1) $$E_{PE}\left(\frac{mg\;PE}{g_{biomass}}\right)=\left(\frac{{OD}_{562}-2.41∙PC-0.849∙APC}{9.62}\right)∙\frac{V_{sample}\left(mL\right)}{m_{biomass}\left(g\right)}$$

(2) $$PC=\frac{{OD}_{615}-0.474∙{OD}_{652}}{5.34}$$

(3) $$APC=\frac{{OD}_{652}-0.208∙{OD}_{615}}{5.09}$$

where OD represents the optical density at the wavelength indicated by the subscript (in nm), PC is the phycocyanin concentration, and APC is the concentration of allophycocyanin.

An external water circulation circuit controlled the temperature in the ultrasonic bath, and the pH was adjusted by adding NaOH 0.05 M or HCL 0.05 M to the extraction media. As a control, the freeze-thaw technique was employed. Dry biomass of *Porphyridium* sp. (100 mg sample) was resuspended in 10 mL of 0.1 M phosphate buffer solution and subjected to repeated eight freeze-thaw cycles with liquid nitrogen to release PE. The cell debris was removed by centrifugation at 5000 rpm for 10 min.

### 2.3 Ionic liquid recovery

The ILs were recovered by dialysis using a 14 kDa molecular weight cut-off membrane. Firstly, the membrane was conditioned by soaking at 40 °C in a distilled water bath for 1 h, with continuous, gentle stirring. The membrane was then submerged into a stirred bath containing an ethanol/water mixture (50 % v/v) at 40 °C. Finally, it was immersed overnight in deionised water under continuous stirring to remove ethanol [42]. The ILs recovery process consisted of three dialysis cycles of 4 h according to the following sequence [42]: first, the crude extract, containing ILs, water, and phycobiliproteins was loaded inside the membrane with deionised water (at a 1:4 crude extract: water volume ratio) for 4 h with continuous stirring, allowing the ILs to permeate through the membrane. Then, the loaded membrane was placed in fresh deionised water, repeating the operation three times. The water phases containing ILs were collected, and the water was partially removed. The remaining water was measured by Karl Fischer analysis using a compact volumetric V20S titrator (Mettler-Toledo LLC. Columbus, OH, USA). Finally, the recovered IL was analysed by attenuated total reflection–Fourier transform infrared spectroscopy (ATR-FTIR) in an ATI Mattson Infinity Series FTIR spectrometer (Mattson Instruments. Madison, WI, USA) to check its purity.

### 2.4 Experimental design

A factorial design of experiments and response surface methodology were used to study and optimise the effect of four independent variables (factors) and their interactions on the extraction of PE. A. The studied dimensionless factors were biomass concentration in the solvent mixture (X_BM_), ionic liquid concentration (X_IL_), [Emim][EtSO_4_]/[Bmim][EtSO_4_] mass ratio (X_E/B_), and ultrasonication time (X_t_). Factors and levels were selected according to previous studies [42]. The chosen levels are summarised in Table 1.

**Table 1:** Correspondence between coded (dimensionless) and actual values of the experimental factors selected for the factorial design.

Factor		Level		
		−1	0	1
Biomass concentration (mg·mL^−1^)	X_BM_	10	35	60
Ionic liquid concentration (wt%)	X_IL_	0	15	30
[Emim]/[Bmim] mass ratio	X_E/B_	1:0	0.5:0.5	0:1
Extraction time (min)	X_t_	10	20	30

The selected response for the study was the PE extraction yield (E_PE_), measured as mg of PE extracted per gram of biomass (dry basis). A face-centred central composite design comprising sixteen full factorial experiments, six central-point replicates and eight face-centred points (star points) was performed. Experiments were run at random to minimise errors due to possible systematic bias. The significance level for the difference between the means was 95 % (*p*-value ≤0.05), and the statistical analysis was performed by using Statgraphics Centurion v19 software (Statgraphics Technologies, Inc. The Plains, VA, USA).

### 2.5 Characterisation of phycoerythrin

The photoluminescence spectra were recorded at room temperature through an Agilent Cary Eclipse Fluorescence spectrophotometer (Agilent Technologies, Inc. Santa Clara, CA, USA) with Czerny-Turner excitation and emission. The excitation source was an Xe pulse lamp pulsed at 80 Hz. PE's excitation and emission spectrum were recorded at λ_ex_ = 498 nm and λ_em_ = 575 nm, respectively.

SDS-PAGE was carried out on 4–20 % Mini Protean TGX precast gels in a Mini Protean Tetra Cell system (Bio-Rad Laboratories, Inc. Hercules, CA, USA) with Laemmli running buffer. The proteins were stained with Bio-Safe Coomassie (Bio-Rad Laboratories, Inc.) in an orbital shaker at moderate speed at room temperature overnight. The gels were destained using three washing cycles with deionised water. Pre-stained Protein Marker 10–250 kDa (dDBiolab SLU. Barcelona, Spain) was used as the molecular weight standard.

Native-PAGE was carried out on NativePAGE Novex 3–12 % Bis-Tris gel (Thermo Fisher Scientific Inc. Waltham, MA, USA). NativePAGE running buffer and NativePAGE cathode buffer (Thermo Fisher Scientific Inc.) were used in the anode and the cathode, respectively. The proteins were stained and destained using the same protocols described for SDS-PAGE. NativeMark Unstained Protein Standard (Thermo Fisher Scientific Inc.) was used as the molecular weight standard.

## 3 Results and discussion

### 3.1 Experimental design

The aim of using the Factorial Design of Experiments and Response Surface Methodology (RSM) was to predict and optimise the response E_PE_ as a function of the operating conditions used for the extraction process. The experimental design matrix and results are shown in Table 2. All the factors are expressed in coded units within the range [−1, +1].

**Table 2:** Experimental design matrix showing the coded levels of the factors and the observed responses.

X_BM_	X_IL_	X_E/B_	X_t_	E_PE_ (mg·g^−1^)
−1	−1	−1	−1	23.2
1	−1	−1	−1	16.6
−1	1	−1	−1	34.1
1	1	−1	−1	23.8
−1	−1	1	−1	23.1
1	−1	1	−1	16.7
−1	1	1	−1	17.8
1	1	1	−1	24.3
−1	−1	−1	1	25.6
1	−1	−1	1	15.7
−1	1	−1	1	35.2
1	1	−1	1	23.6
−1	−1	1	1	24.7
1	−1	1	1	17.3
−1	1	1	1	23.2
1	1	1	1	21.9
−1	0	0	0	35.8
1	0	0	0	28.9
0	−1	0	0	18.7
0	1	0	0	31.4
0	0	−1	0	33.0
0	0	1	0	23.0
0	0	0	−1	30.3
0	0	0	1	35.1
0	0	0	0	31.8
0	0	0	0	31.5
0	0	0	0	31.9
0	0	0	0	33.4
0	0	0	0	32.0
0	0	0	0	31.1

### 3.2 Statistical analysis and modeling

The analysis of variance (ANOVA) of the results (Table 3) showed that extraction time and all its binary interactions and its quadratic effect were not statistically significant at a 95 % confidence interval within the experimental range studied (10–30 min). The literature has reported that the extraction time is a significant variable affecting the amount of phycobiliproteins extracted. However, these studies are carried out with other extraction solvents on different species of microalgae İlter et al., 2018. As is well known, each microalgae species has a distinct cell wall structure and composition [49,50], which leads to different optimisation parameters. This suggests that the extraction process is very fast, and for a time greater than 10 min, no significant improvements are observed in the response value (E_PE_). In addition, the quadratic effects of the biomass concentration (X_BM_·X_BM_) and the binary interaction between biomass concentration and ionic liquid concentration (X_BM_·X_IL_) were not significant either, in agreement with previous work [42].

**Table 3:** Analysis of variance of the factorial design.

Source of variation	Sum of Squares	DF^a^	Mean Square	F-Value	*p*-value
X_BM_	161.64	1	161.64	24.42	0.0002^b^
X_IL_	160.205	1	160.205	24.2	0.0002^b^
X_E/B_	834.201	1	834.201	12.6	0.0029^b^
X_t_	825.534	1	825.534	1.25	0.2817
X_BM_·X_BM_	0.842564	1	0.842564	0.13	0.7262
X_BM_·X_IL_	11.56	1	11.56	1.75	0.2062
X_BM_·X_E/B_	556.516	1	556.516	8.41	0.011^b^
X_BM_·X_t_	11.594	1	11.594	1.75	0.2055
X_IL_·X_IL_	115.603	1	115.603	17.46	0.0008^b^
X_IL_·X_E/B_	570.025	1	570.025	8.61	0.0103^b^
X_IL_·X_t_	0.003025	1	0.003025	0	0.9832
X_E/B_·X_E/B_	371.128	1	371.128	5.61	0.0318^b^
X_E/B_·X_t_	0.555025	1	0.555025	0.08	0.7761
X_t_·X_t_	236.428	1	236.428	0.36	0.559
Total Error	993.009	15	662.006		
Total	1185.05	29			

a Degrees of freedom.

b Significant factor at >95 % confidence level (*p*-value ≤0.05).

The experimental results were fitted to a second-order mathematical model (Eq. 5) where the independent variables corresponded to the coded values of the factors and the dependent variable to the response.:

(4) $$E_{PE}=31.86\;-\;3.00∙X_{BM}+2.98∙X_{IL}-\;2.15∙X_{E/B}+0.68∙X_{t}+0.85∙X_{BM}∙X_{IL}+1.87∙X_{BM}∙X_{E/B}-0.85∙X_{BM}∙X_{t}-\;1.89∙X_{IL}∙X_{E/B}+0.01∙X_{IL}∙X_{t}+0.19∙X_{E/B}∙X_{t}+0.57∙X_{BM}^{2}-6.68∙X_{IL}^{2}-\;3.78∙X_{E/B}^{2}+0.96∙X_{t}^{2}$$

The correlation coefficient value (r^2^ = 0.916) suggests the model fits the experimental results satisfactorily because all the predicted response values lay within the 95 % confidence level (Fig. S1, supplementary material).

**Fig. 1:**
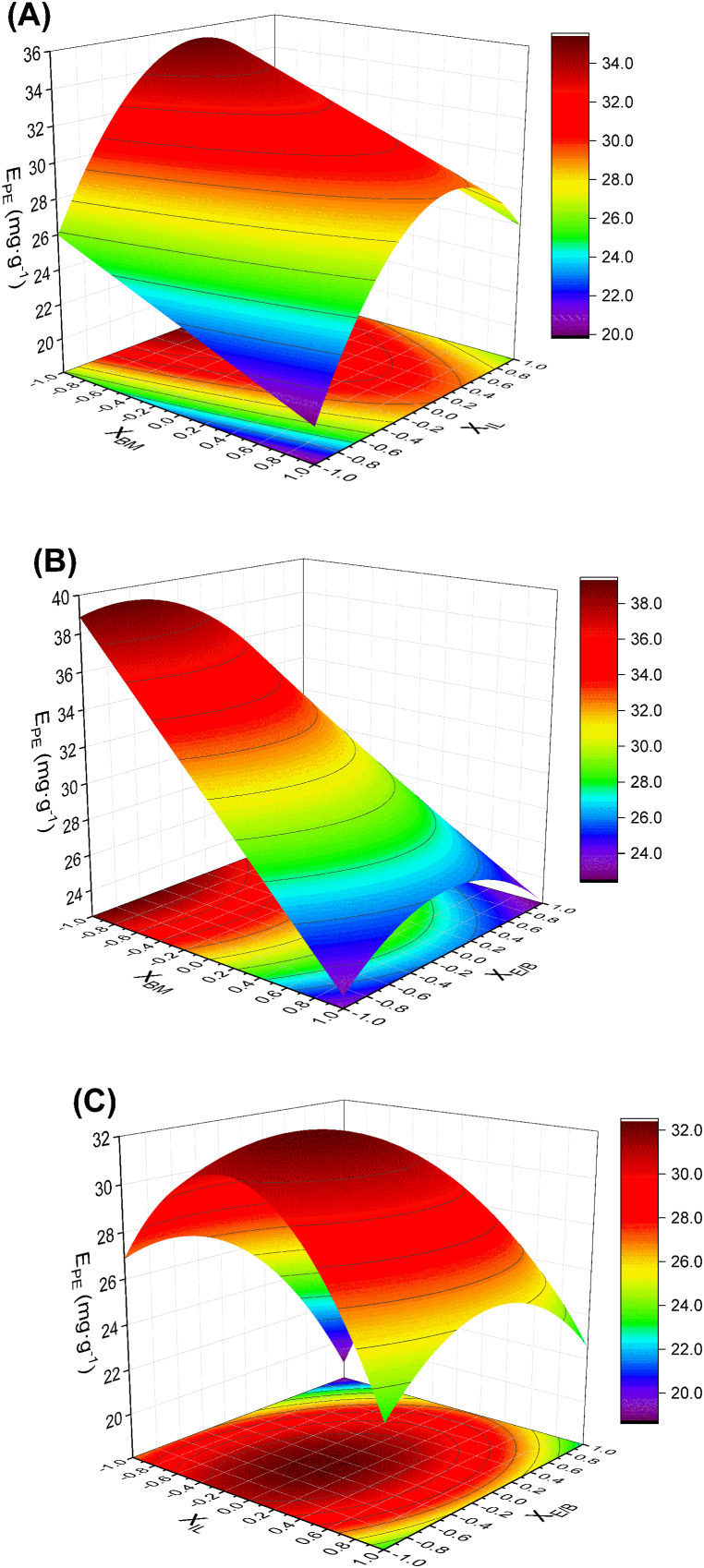
Response surfaces of the extraction yield of PE (E_PE_).

Fig. 1 shows three response surfaces predicted by the model. The selected response (E_PE_) was plotted as a function of two of the significant variables (X_BM_, X_IL_ or X_E/B_), with the third one in its central coded value (0).

### 3.3 Effect of operating conditions on PE extraction yield

**Fig. 2:**
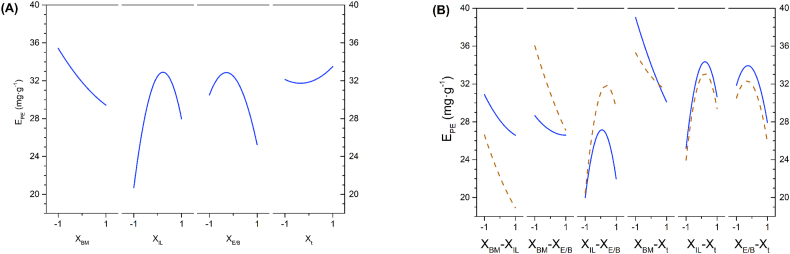
(A) Main effect of the factors on E_PE._ (B) Two-factor interaction on E_PE_ (continuous blue line: +1 level of the second factor, dashed red line: −1 level of the second factor). (For interpretation of the references to colour in this figure legend, the reader is referred to the Web version of this article.)

Fig. 2A shows the main effects of E_PE_. Unlike X_BM_, which shows a linear trend consistent with its non-significant quadratic effect, X_IL_ and X_E/B_ display a distinct curvature with a maximum close to their central values. The observed curvature corroborates that the quadratic effects of both factors (X_IL_·X_IL_ and X_E/B_·X_E/B_) were significant. Fig. 2B represents the binary interactions between the factors. Each pair of curves was obtained using the coded model where the first factor acts as a variable and the second factor is fixed at +1 (continuous-blue line) and −1 (red-dashed line) levels, respectively. Non-parallel curves indicate that binary interactions between two factors are significant. Because time is not a significant factor in the factorial design, the pairs of significant interactions are X_BM_-X_E/B_ and X_IL/W_-X_E/B_, as was also confirmed by the ANOVA (Table 3).

#### 3.3.1 Biomass concentration (X_BM_)

A clear negative trend exists between biomass concentration and PE extraction yield (Fig. 2A). The higher E_PE_ was achieved at the lowest biomass concentration, decreasing its value as biomass concentration increased. This effect is particularly notable when employing exclusively [Emim][EtSO_4_] compared to using only [Bmim][EtSO_4_] (Fig. 2B). Therefore, when each ionic liquid was applied individually, the highest E_PE_ was achieved with [Emim][EtSO_4_] at the lowest biomass concentration. At high biomass concentrations, solvent availability is low, and the phycobiliprotein reaches its partition equilibrium value, preventing the mass transfer between the biomass and solvent mixture from progressing further. However, since the diffusion is driven by the concentration gradient of PE between the biomass and the solvent mixture, the extraction process must be hindered at very low biomass concentration, i.e., the dilution of the system is high. Since this effect was not observed, additional extraction experiments were performed at biomass concentrations below 10 mg mL^−1^ (the lower level in the factorial experimental design). The values of E_PE_ were kept approximately constant, down to a biomass concentration of 2.5 mg mL^−1^, dramatically dropping at a lower concentration (Fig. S2, supplementary material). These results confirmed that no further significant improvement in E_PE_ can be achieved outside the experimental biomass concentration range.

#### 3.3.2 Ionic liquid concentration (X_IL_)

The yield of phycoerythrin extraction increased with rising ionic liquid concentration, reaching a maximum when the concentration slightly exceeded the central value of the experimental range (Fig. 2A). However, at higher ionic liquid concentrations, the extraction yield declined rapidly. As observed, in experiments where the solvent was distilled water (−1 coded value X_IL_ experiments), E_PE_ was lower than those where the solvent was composed of ILs (Table 2, Fig. 1, Fig. 2, Fig. 4) or in the control extraction experiments with buffers solutions (Fig. 4). This agrees with Huschek et al. [51] work, which observed that freeze-dried isolated PE was not soluble in distilled water. However, the PE was effectively re-dissolved in a buffer solution up to a concentration of 3 mg mL^−1^ [51]. Moreover, it has been corroborated in the literature that ionic liquids enhance PE solubility in the aqueous solvent due to the salting-in effect, thereby improving phycoerythrin extraction [40]. However, the decrease beyond the peak yield at high IL concentrations may be likely caused by strengthened ionic forces at higher ionic liquid concentrations. Increased ionic strength stabilises the phycobilisome [52], making phycoerythrin extraction challenging due to its structure and the thylakoid membrane. Moreover, high IL concentration increases the pH, showing a decay in E_PE_ above pH 7.5, as explained in the following sections.

#### 3.3.3 [Emim][EtSO_4_]/[Bmim][EtSO_4_] mass ratio (X_E/B_)

Because of its shorter alkyl chain and, thus, lower hydrophobicity, the use of [Emim][EtSO_4_] as sole IL showed better PE extraction performance than [Bmim][EtSO_4_] (Fig. 2A). This was corroborated in a previous screening of several ILs (Figs. S5 and S6, supplementary material), which also results agree with Martins et al. [40], who observed that ILs with the largest alkyl chain are more hydrophobic and less affine to the surface of the PE, resulting on less solubilisation effect, and therefore presenting lower extraction capacity [40]. Consequently, the enhanced extraction efficiency lacked significance at high biomass concentrations, while the extraction yield notably decreased with an increase in the amount of [Bmim][EtSO_4_] in the mixture at low biomass concentrations.

The interaction biomass concentration and [Emim][EtSO_4_]/[Bmim][EtSO_4_] mass ratio was significant, as shown in Fig. 2B, where the positive impact on E_PE_ of using [Emim][EtSO_4_] and low biomass concentration was confirmed. Consequently, the enhanced extraction efficiency lacked significance at high biomass concentrations, while the extraction yield notably decreased with an increase in the amount of [Bmim][EtSO_4_] in the mixture at low biomass concentrations. The interaction between ionic liquid concentration and ionic liquids mass ratio also significantly influences (Fig. 2B). Thus, both types attained comparable low E_PE_ at low ionic liquid concentrations. Moreover, the beneficial impact on phycoerythrin extraction yield with increasing the concentration of each liquid ionic was also observed in Fig. 2B, although more pronounced in the case of [Emim][EtSO_4_].

A mixture of [Emim][EtSO_4_] and [Bmim][EtSO_4_] in the extraction media produced a significantly higher PE yield compared with the use of each IL separately (Fig. 2A). The better results obtained with the mixture of both ILs were due to the presence of the more hydrophobic [Bmim][EtSO_4_] that can form a two-phase system with water, resulting in less depletion of water molecules from the protein surface and, hence, increasing protein solubility [43]. Besides, the presence of [Bmim][EtSO_4_] in the solvent may favour the dissolution of the lipid wall of the thylakoid membrane, where the phycobilisome is located, making the release of PE easier than in the absence of that IL.

#### 3.3.4 Extraction time (X_t_)

No statistically significant differences in E_PE_ were observed (*p*-value = 0.2817) using different extraction times (Table 3). Experimentally, the solvent could extract most of the PE for a time equal to or greater than 10 min. That result implies that PE mass transfer between the cell matrix and the bulk solvent mixture is very fast, and the extraction process was controlled by the partition equilibrium within the experimental time range studied. This agrees with the fact that the protein extraction yield increases quickly at the early stage of UAE followed by a slower increase until 30 min [39]. Compared with other studies for optimisation of PBP extracted amount through UAE + ILs, the present work considerably reduces extraction time (up to 3-fold reduction) [29,42].

### 3.4 Optimisation

The PE extraction yield (E_PE_) was optimised by applying RSM using the mathematical model (Eq. (3)) describing the process. Table 4 shows the optimal conditions and the predicted maximum value for E_PE_.

**Table 4:** Coded and actual optimum values of the factors maximising the response (Eq. (4)).

	Optimum values	
Factor	Coded	Actual
X_BM_	−1.00	10.0 mg mL^−1^
X_IL_	0.24	18.6 wt%
X_E/B_	−0.57	0.78/0.22
X_t_	1.00	30 min

To validate the model and check the predicted PE maximum yield (39.2 ± 2.6 mg g ^−1^), three replicates were performed at the optimum values of the factors shown in Table 4. Under these conditions, the experimental E_PE_ was 38.8 ± 1.5 mg g^−1^, showing no significant differences (*p*-value ≤0.05) compared to the predicted result, thus corroborating the model's accuracy.

**Fig. 3:**
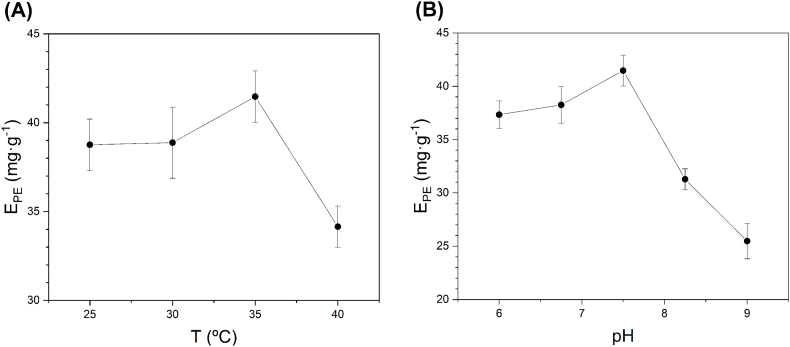
Effect of temperature (A) and pH (B) on E_PE_. All experiments were performed at the optimum conditions from the RSM analysis (BM = 10 mg mL^−1^, IL = 18.6 wt%, E/B = 0.78/0.22, t = 10 min).

Next, the effects of temperature and pH were studied at the optimised conditions from the factorial design (Table 4), except for extraction time, where 10 min was used since the effect of this factor was not significant. Firstly, four temperature values were selected (25, 30, 35 and 40 °C), keeping the pH of the solvent at 7.5 ± 0.01. Each experiment was carried out in triplicate, obtaining the results depicted in Fig. 3A. As observed, the maximum PE extraction yield (41.5 ± 1.5 mg g^−1^) was reached at 35 °C. Secondly, the pH was studied following an analogous procedure, where the temperature was fixed at 35 °C and selecting pH values of 6.00, 6.75, 7.50, 8.25 and 9.00. The highest PE extraction yield (41.5 ± 1.5 mg g^−1^) was achieved at pH = 7.50, as seen in Fig. 3B. The results evidenced an additional increase in E_PE_ by 7 % concerning the experimental value achieved at the optimum conditions resulting from the factorial design, performed at 25 °C and pH 7.5.

**Fig. 4:**
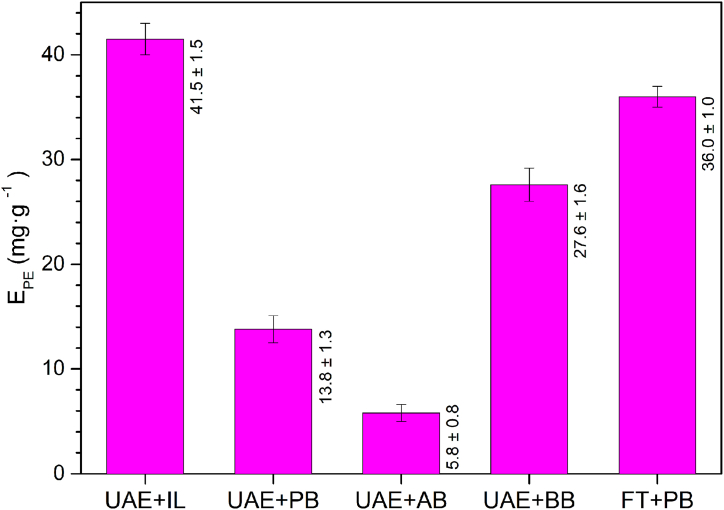
Comparison between E_PE_ values under optimal conditions applying ultrasound-assisted extraction (UAE) or freeze-thaw (FT) methods for cell disruption combined with different aqueous extraction mixtures (IL: ionic liquid mixture optimised in this work, PB: 0.1 M phosphate buffer at pH 7.0; AB: 0.1 M acetate buffer at pH 5.2; BB: 0.1 M borate buffer at pH 9.0). All experiments were performed at 35 °C for 30 min, using a biomass concentration of 10 mg mL^−1^.

#### 3.4.1 Effect of temperature

As observed experimentally (Fig. 3A), the temperature positively affected E_PE_ until it reached its maximum value at 35 °C. That is because PE partition equilibrium and diffusion improve as the working temperature increases since they are endothermic processes that enhance PE extraction. However, at temperatures above 35 °C, PE extraction values decreased due to the degradation of bilin-type chromophores present in PE [53].

#### 3.4.2 Effect of pH

The pH also significantly influenced E_PE_, showing a maximum value of around 7.5 (Fig. 3B). The reason is the balance between the two effects. On one hand, increasing the pH of the medium keeps the acidic amino acids negatively charged and basic amino acids are progressively transformed from their cationic form into their neutral form, increasing the net negative charge of PE, favouring the interaction with the solvent, and hence, the extraction process too [40,54]. However, an increase in pH above 7.5 leads to the degradation of the chromophores, as described by the effect of temperature [53].

#### 3.4.3 Comparison with standard extraction procedures

To contrast the efficiency of the optimised UAE + ILs of PE developed in this work, a comparison with UAE combined with several common extraction solvents (0.1 M acetate buffer at pH 5.2, 0.1 M phosphate buffer at pH 7.0 and 0.1 M borate buffer at pH 9.0) was carried out. In addition, the above UAE-based methods were compared with the extraction using freeze-thaw followed by 0.1 M phosphate buffer extraction at pH 7.0 was performed (Fig. 4).

The UAE + IL extraction process method, developed and optimised in this work, remarkably enhances the PE extraction yield (66.7 %, 86.0 % and 33.5 %) compared to the UAE method combined with phosphate, acetate, and borate buffer solutions, respectively. Likewise, UAE + IL achieves an extraction improvement of 13,1 % compared with using phosphate buffer combined with the freeze-thaw method. Other studies reported in the literature do not show a significant difference in the extracted amount of PBPs between the UAE and the freeze-thaw method [55] or require combining other extraction techniques. Therefore, in this work, a new effective variant of ultrasound-assisted extraction of PE from Porphyridium sp was developed, and the operating conditions were optimised using the simultaneous combination of ILs, which has not been documented previously, resulting in a significant increase in the E_PE._

### 3.5 Ionic liquid reuse

**Fig. 5:**
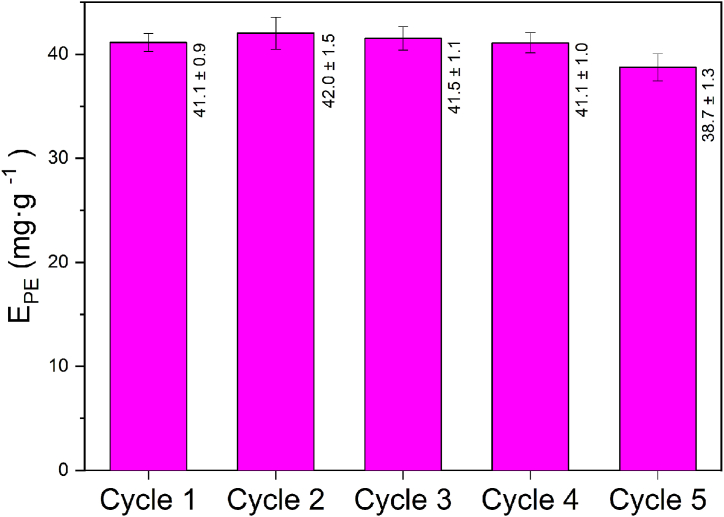
Cycles of reuse of ILs for the extraction of PE.

[Emim][EtSO_4_] and [Bmim][EtSO_4_] were recovered using a dialysis-based procedure and reused for five extraction cycles. Fig. 5 shows that the extraction yield in the fifth cycle was 94.2 % of that obtained in the first extraction, where fresh ILs were used. The IL integrity in each extraction cycle was analysed by ATR-FTIR (Fig. S3, supplementary material). The ILs can be reused successfully, showing the same characteristic IR spectrum as the fresh IL, except the peak centred around 3500 cm^−1^ corresponding to the water remaining in the recovered IL.

The structural integrity of the chromophore of the extracted PE was evaluated by photoluminescence assay (Fig. S4, supplementary material). The excitation spectrum (Fig. S4A) resembles the absorption spectrum at 545 nm and 565 nm and the characteristic shoulder of PE at 498 nm [56]. The emission spectrum of the extraction with fresh and reused ILs shows a clear fluorescence emission peak at 575 nm and a secondary weak peak at 620 nm, typical of PE fluorescence [57]. Therefore, emission spectra of fresh (cycle 1) and reused ILs (cycles 2–5) resembled that of the PE standard, which corroborated the presence and maintenance of the PE structure and the chromophore stability by the similarity of the spectra (Fig. S4B).

**Fig. 6:**
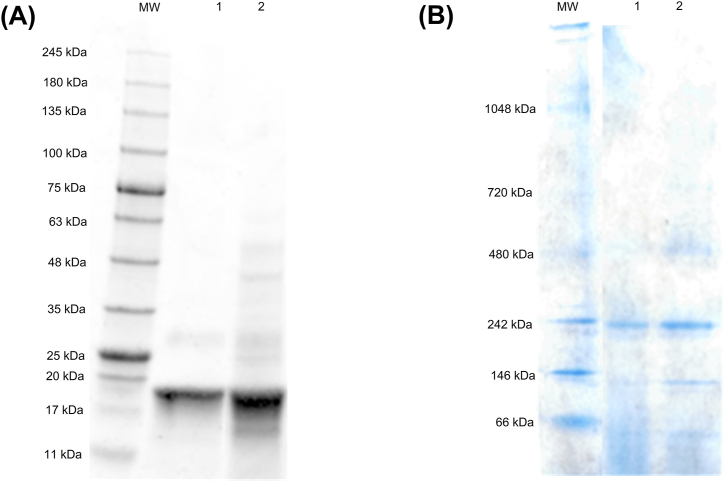
(A) SDS-PAGE of molecular weight marker (MW), PE standard (1) and UAE-ILs extract (2). (B) Native-PAGE of molecular weight marker (MW), PE standard (1) and UAE-ILs extract (2).

To evaluate the protein purity of the extracted obtained, SDS-PAGE and Native-PAGE assays were carried out (Fig. 6). Due to their different characteristic bands, PE, other PBP and the main contaminant proteins appear in the extract. As observed in Fig. 6A, PE was identified in the extract due to the presence of its α and β polypeptide chains of approximately 17–20 kDa and γ polypeptide with an apparent molecular weight of 30–33 kDa and its whole structure was corroborated by the presence of the band of 242 kDa (Fig. 6B).

Therefore, the high price of the ILs, which constitutes a bottleneck for their use as an extraction agent, can be attenuated because of their excellent behaviour upon reusability.

## 4 Conclusions

This work developed and optimised an efficient and sustainable method for extracting PE using conventional ultrasound-assisted extraction coupled with a novel mixture of ionic liquids. The effect of biomass concentration (BM), ionic liquid concentration (IL), ionic liquid composition (E/B), extraction time (t), temperature (T) and pH were studied, and the significance of each one was determined. The maximum PE extraction yield (41.5 ± 1.5 mg g^−1^) was achieved at BM = 10 mg mL^−1^, IL = 18.8 wt%, E/B = 0.78/0.22, t = 10 min, T = 35 °C and pH = 7.5. Moreover, two additional variables were optimised, increasing the amount of PE extracted compared with several common solvents in UAE and the freeze-thaw technique. This work also proves that the ILs can be successfully recovered and reused in five consecutive extraction cycles with a loss in PE extraction yield of <6 % when using fresh ILs. In addition, PE's conformational structure and chromophore integrity were unaffected by using reused ILs.

## Funding

This work was supported by the 10.13039/501100004837Ministerio de Ciencia e Innovación (PID2020-114943RB-I00), and the 10.13039/501100008441CYTED Program (RENUWAL network, 320RT0005).

## CRediT authorship contribution statement

**Alejandro Piera:** Formal analysis, Investigation, Writing – original draft. **Juan J. Espada:** Conceptualization, Supervision, Writing – review & editing. **Victoria Morales:** Writing – review & editing. **Rosalía Rodríguez:** Formal analysis, Writing – review & editing. **Gemma Vicente:** Conceptualization, Funding acquisition, Supervision, Writing – review & editing. **Luis Fernando Bautista:** Conceptualization, Funding acquisition, Supervision, Writing – review & editing.

## Declaration of competing interest

The authors declare that they have no known competing financial interests or personal relationships that could have appeared to influence the work reported in this paper.
