# Supplementary material for: Optimised phycoerythrin extraction method from Porphyridium sp. combining imidazolium-based ionic liquids
Source: Heliyon. 2024 Jul 20;10(14):e34957. doi: 10.1016/j.heliyon.2024.e34957 (PMC11325355; doi:10.1016/j.heliyon.2024.e34957)
Supplement: Multimedia component 1 [file mmc1.docx]

**SUPPLEMENTARY MATERIAL**

**Optimised phycoerythrin extraction method from *Porphyridium* sp. combining imidazolium-based ionic liquids.**

Alejandro PIERA ^a^, Juan J. ESPADA ^b^. Victoria MORALES ^a^, Rosalía RODRÍGUEZ ^b^, Gemma VICENTE ^b,c^, Luis Fernando BAUTISTA ^a,c,*^

^a^ Department of Chemical and Environmental Technology. ESCET, Universidad Rey Juan Carlos, 28933, Móstoles, Madrid, Spain.

^b^ Department of Chemical, Energy and Mechanical Technology. ESCET, Universidad Rey Juan Carlos, 28933, Móstoles, Madrid, Spain.

^c^ Instituto de Tecnologías para la Sostenibilidad, Universidad Rey Juan Carlos, 28933, Móstoles, Madrid, Spain.

* Corresponding author: Phone: +34 914888501

E-mail: [fernando.bautista@urjc.es](mailto:fernando.bautista@urjc.es)





**Figure S1.** Experimental versus predicted values of the extraction yield of PE (E_PE_). Red-dashed lines show the confidence interval at a 95% confidence level.





**Figure S2.** Effect of biomass concentration on phycoerythrin extraction yield (Ionic liquid concentration: 18.6 wt%, [Emim]/[Bmim] mass ratio: 0.78/0.22, extraction time: 10 min, temperature: 25ºC, pH: 7.5).





**Figure S3.** ATR-FTIR analyses of reused ILs.







**Figure S4.** (A) Fluorescence absorption spectra of standard (red line) and extracted (blue line) PE. (B) Fluorescence emission spectra (λ_ex_ =498 nm). of standard and extracted phycoerythrin in all extraction cycles performed with reused ILs. All spectra were normalised since these were used on a comparative basis.





**Figure S5.** Comparison between E_PE_ values obtained using different aqueous solutions of imidazolium family ionic liquids. E: [Emim][EtSO_4_]; B: [Bmim][EtSO_4_]; H: [Hmim][EtSO_4_]; O: [Omim][EtSO_4_])





**Figure S6.** Color comparison between aqueous solutions of imidazolium family ionic liquids: (A) [Emim][EtSO_4_]; (B) [Bmim][EtSO_4_]; (C): [Hmim][EtSO_4_]; and (D) [Omim][EtSO_4_].
